# Dataset for measuring the conceptual understanding of optics in Rwanda

**DOI:** 10.12688/f1000research.53135.2

**Published:** 2022-05-10

**Authors:** Kizito Ndihokubwayo, Michael Ralph, Irénée Ndayambaje, Jean Uwamahoro

**Affiliations:** 1 African Center of Excellence for Innovative Teaching and Learning Mathematics and Science (ACEITLMS), University of Rwanda College of Education (URCE), Kayonza, P.O Box 55 Rwamagana, Rwanda; 2 Department of Educational Psychology, University of Kansas, Joseph R. Pearson Hall, Rm. 621, 1122 West Campus Rd. Lawrence, Kansas, 66045-3101, USA

**Keywords:** light phenomenon, optics, conceptual understanding, Rwandan students

## Abstract

This dataset is an accumulation of data collected to test Rwandan physics students’ conceptual understanding of light phenomena and to assess instructional tools for active learning of optics. We collected and analysed data from 251 grade 11 (senior 5) students using our Light Phenomena Conceptual Assessment (LPCA) tool and from 136 grade 10 (senior 4) students using Geometric Optics Conceptual Understanding Test (GOCUT) in 2019. Before collecting data, we designed and validated LPCA and GOCUT, and tested their reliability. Data were collected before and after students learnt about the unit of light. Both day and boarding schools in rural and urban areas were included in our sampling. Data collected were test scores from students after performing a 30-item LPCA test or 25-item GOCUT test in 40 minutes. The data may be reused to extend  students' understanding of optics concepts through item analysis, analysis of school characteristics such as location and school type, or by analysing students' characteristics such as subject combinations.

## Introduction

Assessment inventories data provide insights into the classroom atmosphere and show students’ progress in grasping certain concepts, and these are essential for teachers, educationists, educational evaluators, and researchers. Such inventories may be used to test students’ understanding of a certain concept or may be used to test the effectiveness of a particular teaching approach or instructional tool. This dataset is an accumulation of data collected for the first author’s doctoral research project “ *Assessment of Instructional Tools for Active Learning of Optics at Advanced Level Secondary Schools in Rwanda*”. ^1– 6^ In this project, there was a need to first assess students’ conceptual understanding of light phenomena and, second, to assess the effectiveness of instructional tools (such as University of Colorado Boulder’s interactive PhET simulations, and YouTube videos) to improve the learning of optics. Thus, this article presents data from two different inventories or tests that were designed. (i) the Light Phenomena Conceptual Assessment (LPCA) and (ii) Geometric Optics Conceptual Understanding Test (GOCUT). Both these datasets are useful to researchers that will use LPCA, or GOCUT data, or to those who want to understand Rwandan physics students’ performance. This will enable researchers to reanalyse the data in different contexts, such as item analysis theory, comparing school characteristics such as students’ performance in day schools compared to boarding schools, comparing rural schools to urban schools, analysing subject combinations, etc. In this vein, LPCA data are discussed in detail to guide research practitioners on how students’ performance and test item performance-related data are analysed.

The study describing the development of the LPCA tool and its implementation was published in Physics Education (PED) ^6^ and the LPCA study instrument is available on protocols.io and Physport platform. The LPCA is a conceptual understanding test composed of 30 items addressing geometric and physical optics. It was designed based on students’ misconceptions related to the everyday understanding of light phenomena. The data connected to this tool are available in *Underlying data* ^7^ and were listed and analysed in a Microsoft Excel file titled ‘Pre-Post-Test LPCA Data - Senior 5 Rwandan physics students’. This file contains three sheets; the first sheet presents the pre-test data, the second sheet presents the post-test data, while the third sheet contains filtered data (students who performed both the pre- and post-test). The data comprises various students’ backgrounds; rural and urban schools, boarding and day schools, and different subject combinations (see Table 1 in Methods section).

**Table 1.** Characteristics of data for the Light Phenomena Conceptual Assessment (LPCA) implemented at S5 or Grade 11. Note: PCM: Physics-Chemistry-Mathematics, PCB: Physics-Chemistry-Biology, MPC: Mathematics-Physics-Computer science, MPG: Mathematics-Physics-Geography

s/n	School	Class	Combination	No of students at Pre-test	No of students at Post-test	Filtered
1	School 1	3	PCM, PCB, MPC	23, 41, 43	25, 43, 39	19, 39, 36
2	School 2	1	PCB	26	30	23
3	School 3	1	MPG	32	31	30
4	School 4	1	MPG	16	19	16
5	School 5	1	PCB	27	24	23
6	School 6	1	PCB	44	40	38
7	School 7	1	MPG	19	16	16
8	School 8	1	MPG	12	11	11
Total				283	278	251

The study describing the development and implementation of the GOCUT tool was published in the African Journal of research in Mathematics, Science and Technology education (AJRMSTE). ^3^ The revised protocol where rote learning-related items were removed, is also available in. ^2^ The GOCUT is a conceptual understanding test composed of 25 items of geometric optics. It was designed based on various existing inventories. The data connected to the GOCUT study are available in *Underlying data* ^8^ and were listed and analysed in a Microsoft Excel file titled ‘Pre-Post-Test GOCUT Data - Senior 4 Rwandan physics students’. This file contains seven sheets; the first sheet introduces the data collected, while other sheets present pre-test and post-test data for three groups of instructional tools of intervention (control group, PhET simulations group, and YouTube videos group). The data comprises various students’ backgrounds; rural and urban schools, boarding and day schools, and different subject combinations (see Table 2 in Methods section).

**Table 2.** Characteristics of data from the Geometric Optics Conceptual Understanding Test (GOCUT) implemented at S4 or Grade 10. Note: PCM: Physics-Chemistry-Mathematics, PCB: Physics-Chemistry-Biology, MPC: Mathematics-Physics-Computer science, MPG: Mathematics-Physics-Geography

School	School location	School type	Subject combination	Teaching intervention	No of students at Pre- and Post-test
School 1	Urban	Boarding	PCM	YouTube videos	30
School 1	Urban	Boarding	PCB	PhET simulations	36
School 2	Urban	Day	PCB	Traditional methods	29
School 3	Urban	Boarding	MPG	Traditional methods	16
School 4	Urban	Day	MPG	PhET simulations	9
School 7	Rural	Boarding	MPG	YouTube videos	16
Total					136

## Methods

### Data collection

*LPCA*

**Figure 1.**
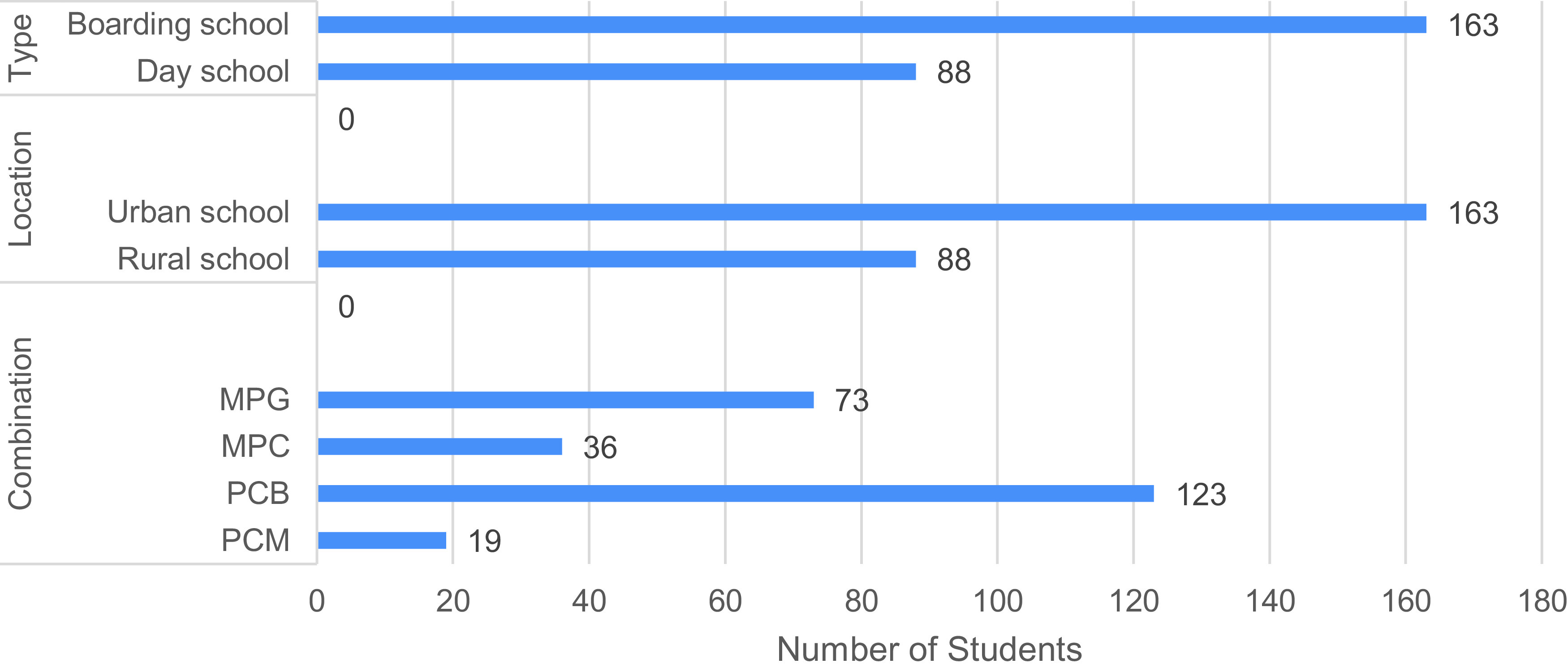
Number of students who carried out the Light Phenomena Conceptual Assessment (LPCA), according to type and location of school, and subject combination characteristics. PCB: Physics-Chemistry-Biology, MPG: Mathematics-Physics-Geography, PCM: Physics-Chemistry-Mathematics, MPC: Mathematics-Physics-Chemistry.

A total of eight Rwandan secondary schools were involved in the study. We selected two districts in Kigali city, and two districts in the rural Eastern Province. We listed the schools in those four districts, and chose two schools from each district that accommodated physics in their subject combinations. School characteristics, location, and type of school (School 1 to School 4 are from Kigali, while School 5 to a School 8 were from the eastern province, see Figure 1) were considered during the selection process. These school characteristics, location, and type of school were considered during the selection process so as to include a diverse group of students and to avoid any potential sources of bias.

We employed a pre- and post-test design ^9^ to collect the data for measuring students conceptual understanding of optics-related concepts. The LPCA was administered twice to the students via paper form, before and after learning about the unit of light in senior-5. ^10^ A total of 251 students from grade 11 or senior 5 (S5) were considered the final sample after removing those who sat for pre-test and missed post-test, and vice versa (see Table 1). The methods for the data coding are presented in the Analysis section. These students had no other teaching interventions offered apart from usual teaching.

*GOCUT*

The boarding and day secondary schools chosen to be involved in the GOCUT were the same as for the LPCA (schools from rural areas were sampled from Eastern Province, while those from urban areas were sampled from Kigali city). However, three schools were excluded due to ineffectiveness of implementing the designed intervention. Thus, researchers were not able to implement the intervention at these schools. Students were from grade 10 or senior 4 (S4), with various subject combinations. PCB: Physics-Chemistry-Biology, MPG: Mathematics-Physics-Geography, PCM: Physics-Chemistry-Mathematics. Table 2 displays characteristics of school and students in which the instructional tools were implemented and GOCUT was administered.

Teaching interventions of PhET simulations and/or videos compiled on YouTube were offered (see Table 2) to the students. Details of the YouTube videos, including the names of any companies/institutions responsible for creating the materials are available in ^3^ p. 257). GOCUT was administered twice to the students via paper form, before and after learning about geometric optics via the teaching interventions in senior-4. ^10^ A total of 136 students from grade 10 or senior 4 (S4) were involved in the study (see Table 2).

The data were initially (pre-test) collected in January 2019 and finally (post-test) at the end of March 2019. The answer choices for GOCUT are A, B, C, and D. These choices measure the students’ conceptual understanding of optics, where one is stem (correct answer) while other three choices are distractors (wrong answers). Where the student did not answer, N is coded, while where the student answered more than one answer, T is coded. For the drawing question (item 13), C was coded for students who correctly drew, while W was coded for those who wrongly drew. For the explanatory question (item 9), the extended explanation was provided in the column after AH, after the drawing question.

### Analysis

This section presents the step-by-step analysis of the LPCA data. We took the case of the first inventory (light phenomena conceptual assessment, LPCA) to extend the description of analysis to help research practitioners in educational research get insight into performance and conceptual understanding test analysis. Please note that unlike the LPCA data file, the file for the GOCUT does not provide accumulated or detailed analysis. Nevertheless, LPCA and GOCUT are similar in manner; their data were recorded and arranged in the same way, so the explanation of how we analysed LPCA data may be used to analyse the GOCUT data.

We used Microsoft Excel 2016 to analyse the data. Since the LPCA test was a multiple-choice test (except for item 11 which requests a supporting explanation), each item has four choices—from A to D. We recorded this data in an Microsoft Excel sheet by putting an assigned letter to each item (A, B, C, or D). Where a student assigns more than one answer, we recorded “T” while where the student selects nothing or skips the question; we recorded “N.”

The first analysis was to use “COUNTIF” function to count the number of students who answered each letter; the sum should be the total number of students (see, for example, in pre- or post-test sheet, column F, row 4-10). The second analysis was to mark students by giving a score of “1” to everyone who answered each item correctly (who chose the right answer) and by giving a score of “0” to those who selected the wrong answer, did not answer, or selected more than one answer. We use “IF” and “EXACT” functions (see, for example, column AM, row 15). After computing these functions for each student, we summed the total scores for each student (see column BR) and the corresponding percentage scores (see column BS). These percentage scores show the students’ performance (scores received by every student over the whole LPCA test). A histogram was computed to check the normal distribution of the test scores (number of students in each assigned interval of scores, please see column BU-CG). The significance of performance before and after learning optics was computed in the filtered sheet (see column W-AA).

The third analysis was item analysis. See the bottom of “IF” and “EXACT” analysis on row 299 in pre-test sheet, for example. The sum of scores for each item of LPCA was computed to reveal the difficulty of the test. A graph was generated showing all 30 items; among them, some are difficult (performed by few students), and others are easy (performed by most of the students). In other words, it was more difficult to perform well in some of the items, and that these items were answered by fewer students. For this analysis, further analysis may generate a graph showing the answer choice for each item (please refer to the *Underlying data*. ^7^ It shows the number of students who selected every letter for each item. It shows how the correct answer varies from alternative choices and analyses the students’ misconceptions. This figure is generated using the records of the first analysis (counted numbers of answers using “COUNTIF” function).

In the filtered sheet, we have filtered the students who sat for both pre- and post-test. This helps for side-by-side analysis of the results and helps to keep each student’s scores parallel so that the difference between both test scores is clear. It tracks the performance along with both tests, i.e., whether the students performed better in the post-test or the inverse. If it is inverse, analysis of misconceptions and a revisit of the instructions may be further studied (to understand why the student failed after learning, performing even more worse than he/she performed before learning). We have shown how Cohen’s D effect size and Normalised learning gains <g> are computed to measure the impact of instruction (see column W-AA, row 259-269). Effect size is computed by taking the difference of means of post-test and pre-test dividing by the average of standard deviations (see cell Y263). Cohen, ^11^ Sawilowsky, ^12^ and Mangnusson ^13^ interpret “d” of 0.20 as small, 0.50 as a medium, and 0.80 as large. Normalised learning gain <g> is calculated by taking the difference of means of post-test and pre-test, dividing by the maximum mean. The maximum mean is the difference of 100% or highest score and the mean of pre-test scores (check out cell Y264). Hake ^14^ interprets a <g> of <.3 as small, <g> of.3 to.6 as medium, and large and <g> of >.7 as large.

### Validation

The data from both tools are valid and reliable as the tools underwent a rigorous validation and a test-rested reliability was checked before the official use. We first searched the literature for possible misconceptions that students had on the topic of optics and available tests to remedy them. We then drafted questions, using our experiences from the classroom, Rwandan textbooks (in case of LPCA), and existing tests, research articles and textbooks (in the case of GOCUT). We shared the survey questions with four university professors in physics education for content validation (i.e. to check that the questions were testing the real constructs/concepts we intend to evaluate) and to 38 students–selected from two schools from elsewhere, i.e. schools not included in this study—for face validation (i.e. to check the difficulty of questions so as to identify any confusion that may rise). The initial number of questions for each test was above 50 items, after improving them using suggestions from both validators, we reached 30 LPCA items and 25 GOCUT items.

## Ethics statement

The study procedure was approved by the ethical committee in the University of Rwanda College of Education’s research unit and innovation (permit number: 01/P-CE/483/EN/gi/2018). Ethical clearance was provided after reviewing our research proposal. Our data collection involved secondary school students aged between 16 and 23 years old. Parental consent was not obtained for students under 18 (adult age in Rwanda); however, the study was considered low risk. We explained the purpose of our study to teachers and asked teachers, as well as the students, to sign an informed consent form before partaking in our tests and study. We assured them that the voluntary participation and publication of data would not reveal individual participants’ identities. Data were treated confidentially, and we have deleted the students’ names from our data to maintain their anonymity. Since the first protocol (LPCA) was fully designed by authors and the second protocol (GOCUT) was designed based on existing tests, there was no special approval obtained from developers, however, we fully credited their sources and works.

## Data availability

### Underlying data

Mendeley Data: Pre-Post-Test LPCA Data: Senior 5 Rwandan physics students. https://data.mendeley.com/datasets/dbvh59jg7j/1. ^7^

This project contains the following underlying data:

- LPCA.pdf (copy of the light phenomena conceptual assessment (LPCA), an inventory test of 30 items)
- Pre-Post-Test LPCA Data - Senior 5 Rwandan physics students.xlsx (MS Excel file that contains the data)

Mendeley Data: Pre-Post-Test GOCUT Data: Senior 4 Rwandan physics students. https://data.mendeley.com/datasets/mmtpw5nvg3/1. ^8^

This project contains the following underlying data:

- GOCUT.pdf (copy of the geometric optics conceptual understanding test (GOCUT), an assessment test of 25 items)
- Pre-Post-Test GOCUT Data - Senior 4 Rwandan physics students.xlsx (MS Excel file that contains the data)

Data are available under the terms of the Creative Commons Attribution 4.0 International license (CC-BY 4.0).
