# Supplementary material for: Dataset for measuring the conceptual understanding of optics in Rwanda
Source: F1000Res. 2022 May 10;10:679. Originally published 2021 Jul 28. [Version 2] doi: 10.12688/f1000research.53135.2 (PMC9120929; doi:10.12688/f1000research.53135.2)
Supplement: Peer review history

# Peer review: Reviewer response for version 2

**DOI:** 10.5256/f1000research.133699.r137209

**Published:** 2022-05-19

**Authors:** Imelda Kemeza

**Affiliations:** 1 Department of Educational Foundations and Psychology, Mbarara University of Science and Technology, Mbarara, Uganda

Going by the ethics statement:

 The authors committed to the best practices for ethics when working with human subjects in this case parents, however, the involvement of students under 18 (Rwandan adult age) on the pretext of the study being considered of low risk seems to weigh in less as a waiver when students under 18 have not reached the legal age of consent to participate in a research activity. This, too, raises questions on the help to develop the students’ autonomy and what supports communication between the researcher and the students under 18.

 I approve the dataset for measuring the conceptual understanding of optics in Rwanda where it meets with international standards on ethical guidelines.

Are sufficient details of methods and materials provided to allow replication by others?

Partly

Is the rationale for creating the dataset(s) clearly described?

Partly

Are the datasets clearly presented in a useable and accessible format?

Partly

Are the protocols appropriate and is the work technically sound?

Partly

Reviewer Expertise:

Educational Psychology with a bias to psychometrics

I confirm that I have read this submission and believe that I have an appropriate level of expertise to confirm that it is of an acceptable scientific standard.

# Peer review: Reviewer response for version 2

**DOI:** 10.5256/f1000research.133699.r137210

**Published:** 2022-05-12

**Authors:** Hassen Ghalila

**Affiliations:** 1 Department of Physics, Faculty of Sciences, University of Tunis El Manar, Tunis, 1060, Tunisia; 2 LSAMA Laboratory, University of Tunis El Manar, Tunis, Tunisia

I would like to thank the authors for their commitment to this very important issue. Even if they do not fully answer the first question (analyses of the LPCA dataset have already been done in detail in the previous article (Ref.5), it would have been preferable to adapt the manuscript to the results obtained with the supplementary tools used in the GOCUT field), which was also pointed out by the other reviewer, I give my approval for the publication of their article.

Are sufficient details of methods and materials provided to allow replication by others?

Partly

Is the rationale for creating the dataset(s) clearly described?

Partly

Are the datasets clearly presented in a useable and accessible format?

Partly

Are the protocols appropriate and is the work technically sound?

Partly

Reviewer Expertise:

Physics of plasma and spectroscopy

I confirm that I have read this submission and believe that I have an appropriate level of expertise to confirm that it is of an acceptable scientific standard.

# Peer review: Reviewer response for version 1

**DOI:** 10.5256/f1000research.56488.r124417

**Published:** 2022-03-18

**Authors:** Imelda Kemeza

**Affiliations:** 1 Department of Educational Foundations and Psychology, Mbarara University of Science and Technology, Mbarara, Uganda

The authors’ manuscript created a dataset for LPCA and GOCUT for optimized conceptual understanding of light phenomena and also assessed instructional tools for active learning of optics in Physics.

 Major concerns:

- In my view, the manuscript authors punctuated the LPCA analysis with appropriate and relevant explanations. A similar treatment to GOCUT analysis would have informed comparisons and discussion of the dual analyses for a better-positioned dataset.
- In the ethics statement: “Our data collection involved secondary school students aged between 16 and 23 years old. Parental consent was not obtained for students under 18 (adult age in Rwanda); however, the study was considered low risk.” Whether the study was considered low risk, research ethics are clear on what should be done when participants are under-aged. In this case, where some students were aged 16 and 17 years old, an assent and/or consent is deemed fit from the right population.

Minor corrections:

- In the introduction section, third paragraph, second sentence: “The revised protocol where rote learning-related items where removed, is also available in.” Replace the second 'where' with 'were'.
- In the methods section, GOCUT in the second paragraph, the last sentence, change S5 to S4 for consistency in grade choice.

Are sufficient details of methods and materials provided to allow replication by others?

Partly

Is the rationale for creating the dataset(s) clearly described?

Partly

Are the datasets clearly presented in a useable and accessible format?

Partly

Are the protocols appropriate and is the work technically sound?

Partly

Reviewer Expertise:

Educational Psychology with a bias to psychometrics

I confirm that I have read this submission and believe that I have an appropriate level of expertise to confirm that it is of an acceptable scientific standard, however I have significant reservations, as outlined above.

# Response

**Published:** 2022-05-02

**Authors:** Kizito Kizito

**Affiliations:** University of Rwanda, Rwanda

Dear Reviewer,

 Thank you for the prodigious comments provided!

- We could not describe GOCUT analysis methods, but since GOCUT data and LPCA data are presented in similar ways and the same analysis discussed for LPCA would be appropriate for GOCUT. This was also highlighted in the manuscript. Actually, when you click on a cell, you can identify the formula used to compute a certain function.
- Thank you so much for the informative instruction on the ethics statement.
- Regarding the typo in the introduction section, “where” was replaced by “were.”
- Regarding the typo in the method section, S5 was replaced by S4.

# Peer review: Reviewer response for version 1

**DOI:** 10.5256/f1000research.56488.r101516

**Published:** 2021-12-09

**Authors:** Hassen Ghalila

**Affiliations:** 1 Department of Physics, Faculty of Sciences, University of Tunis El Manar, Tunis, 1060, Tunisia; 2 LSAMA Laboratory, University of Tunis El Manar, Tunis, Tunisia

Your manuscript reports on the development and elaboration of a dataset for LPCA and GOCUT to help teachers and the educational system more generally to improve conceptual understanding of optics and associated light phenomena.

**Major corrections**

1. In my sense, a too great part of this manuscript is dedicated to the LPCA dataset. This is not necessary since you have already done this in your previous article (Ref.5). It would have been preferable to develop more analysis on the results obtained with the supplementary tools used in the GOCUT field. This discussion is totally absent in this manuscript, and I think will help to better address the dataset. Figures inside the excel files (only one for GOCUT?) could be used to help the comparison between the different tools employed with their associated results. It seems (after a very brief look at your data) that lessons using YouTube performed better than the other two? Also, it would be helpful to recall the ‘usual teaching’ (content and way of teaching) for the S4 students.
2. Your samples are composed of four groups, one from the rural zones with boarding and day schools and another two from Kigali again with boarding and day schools. I didn’t see any discussion or comparison on the results obtained for these four groups. Does that mean that there is no difference between them?
3. In the dataset of LPCA, question 7 answers A and C are similar? Is it an error or something missing?

**Minor corrections**

1. In the Introduction section: One of the two references 7 and 8 is too much and not necessary.
2. In the Methods section, GOCUT: The second to last paragraph, ‘A total of 136 students from grade 10 or senior 4 (S5) were involved in the study (see Table 2)’. Replace S5 with S4.
3. In the Ethics statement section: ‘Our data collection involved secondary school students aged between 16 and 23 years old.’. Is it 16 or 18 years old?

Are sufficient details of methods and materials provided to allow replication by others?

Partly

Is the rationale for creating the dataset(s) clearly described?

Partly

Are the datasets clearly presented in a useable and accessible format?

Partly

Are the protocols appropriate and is the work technically sound?

Partly

Reviewer Expertise:

Physics of plasma and spectroscopy

I confirm that I have read this submission and believe that I have an appropriate level of expertise to confirm that it is of an acceptable scientific standard, however I have significant reservations, as outlined above.

# Response

**Published:** 2022-05-02

**Authors:** Kizito Kizito

**Affiliations:** University of Rwanda, Rwanda

Dear Reviewer,

 Thank you so much for your insightful comments and suggestions!

- Yes, LPCA was greatly discussed. Both LPCA and GOCUT datasets were described, and the methods used to collect related data were presented. We have clarified in the manuscript that both data sets are similar, although the participants, levels, and tests were different. The data entry was the same. That is why describing the analysis of all of them would be unnecessary. Thus, to avoid duplication of text, we opted to take LPCA as a reference and discuss its analysis procedures. So researchers who want to look at whether GOCUT can use the same analysis methods we presented for LPCA. The figure presented in the GOCUT dataset can be referred to as a plot of others in other sheets since MS Excel formulae are presented in corresponding cells.
- Regarding variables in our sample, the difference between them may be there or not, but this data note is not dedicated to presenting results, discussing, or drawing any conclusion; it just provides room for other researchers to reuse the data. Thus, the collection procedure and proposed data analysis are only presented.
- Regarding question 7 in the LPCA dataset, we could not find a mistake. If you mean answer choices in pre- and post-test, we found them different.
- Regarding the introduction section, since the link for LPCA protocol was provided in the text, reference 7 was deleted.
- Regrading the typo in the methods section, S5 was replaced by S4.
- Regarding the ethical statement section, it is 16 years old.
